# Risk for pediatric intensive care utilization in children born before 30 weeks of gestation: a single-center study

**DOI:** 10.1007/s00431-025-06714-4

**Published:** 2026-01-19

**Authors:** Annemijn C. C. B. Ekelmans, Rosemarie de Ridder, Suzanne W. J. Terheggen, Marieke H. Otten, Anton H. van Kaam, Job B. M. van Woensel, G. Jeroen Hutten, Reinout A. Bem

**Affiliations:** 1 Neonatal Intensive Care Unit, Emma Children’s Hospital, Amsterdam University Medical Centers, University of Amsterdam, Amsterdam, The Netherlands; 2 Department of Pulmonology, Emma Children’s Hospital, Amsterdam University Medical Centers, University of Amsterdam, Amsterdam, The Netherlands; 3 Pediatric Intensive Care Unit, Emma Children’s Hospital, Amsterdam University Medical Centers, University of Amsterdam, Amsterdam, The Netherlands; 4 Amsterdam Reproduction & Development Research Institute, Amsterdam, The Netherlands

**Keywords:** Prematurity, Neonatal intensive care unit, Pediatric intensive care unit, Readmission, Long-term morbidity

## Abstract

Extremely preterm-born infants face critical complications after birth that may necessitate health care utilization later in life, including rehospitalization with admission to the pediatric intensive care unit (PICU). However, their specific risk for PICU readmission after discharge from the neonatal intensive care unit (NICU) is currently unclear. Further knowledge on this topic may aid critical care resource planning and parent counseling. In this study, we aimed to determine the rate of PICU admission in preterm infants born with a gestational age (GA) < 30 weeks, and to identify risk factors associated with PICU admission. This is a retrospective, single-center study including patients between 2016 and 2021. A total of 459 patients were included. Of these, 50 (10.9%) were admitted to the PICU at least once during a follow-up of 3–9 years after NICU discharge. The proportion of children admitted to the PICU varied by GA, ranging from 23.5% at 24 weeks GA to 6.5% at 29 weeks GA. The median (IQR) age of first admission was 4 (3.0–15.5) months. GA < 28 weeks and necrotizing enterocolitis (NEC, grade ≥ 2) were independently associated with an increased risk of PICU admission (OR 2.41, 95%CI 1.22–4.76, *p* = 0.012; and OR 6.20, 95%CI 3.18–12.10, *p* < 0.001, respectively).

*Conclusion*: In our cohort, one in ten patients born < 30 weeks gestation experienced a PICU admission in their early youth after discharge from the NICU. Extreme prematurity and a diagnosis of NEC were associated with PICU admission.

**Table:** 

**What is Known:** • *Survival of preterm infants has increased.* • *Preterm-born children have an increased risk of morbidity, which predisposes them for a higher risk of further health care utilization after neonatal intensive care unit (NICU) discharge.*
**What is New:** • *In our single-center study, approximately one in ten patients born with a gestational age (GA) < 30 weeks are admitted to the pediatric intensive care unit (PICU) in the years following discharge from the NICU.* • *Risk factors associated with PICU admission were extreme prematurity (GA < 28 weeks) and necrotizing enterocolitis diagnosis ≥ grade 2.*

**Supplementary Information:**

The online version contains supplementary material available at 10.1007/s00431-025-06714-4.

## Introduction

Over the last decades, survival of preterm infants in high-income countries has increased substantially [1]. Regardless, many preterm infants face severe complications, such as necrotizing enterocolitis (NEC) and bronchopulmonary dysplasia (BPD), during their stay in the neonatal intensive care unit (NICU). These complications predispose them to long-term additional morbidity and higher risk of necessitating further health care utilization [2, 3].

Long-term morbidity following preterm birth may also increase the risk for admission to the pediatric intensive care unit (PICU). A study from the USA found that approximately 8% of all PICU patients have a history of preterm birth [4], and that these children utilize more acute and chronic resources during their stay than non-premature born children [4, 5]. Previous registry research in England and Wales, involving more than 40,000 preterm infants, found that PICU admission rates vary by gestational age (GA), ranging from 13.6% for infants with a GA < 24 weeks to 3.7% for those with a GA of 31 weeks [2]. Viral respiratory tract infection appears to be an important reason for PICU admission among preterm-born infants [6].

Better insight into PICU utilization in preterm-born children is imperative for appropriate resource and follow-up planning, development of preventive strategies, and counseling of the parents of these children. Yet, up to date, studies analyzing PICU resource use in preterm-born children have been limited to follow-up during the first 2 years of life [2, 5]. Furthermore, data on PICU admission of former preterm infants outside the UK and USA is scarce. Therefore, the aim of this study was to determine the rate of PICU admission(s) following preterm birth before 30 weeks gestation in a Dutch tertiary children’s hospital, and to analyze associated risk factors.

## Methods

This retrospective single-center cohort study included all preterm infants with a GA < 30 weeks admitted to the NICU of the Emma Children’s Hospital at Amsterdam University Medical Center (AUMC) between January 1, 2016, and December 31, 2021. Patients who died during their NICU stay were excluded. PICU admissions were assessed until March 17, 2025, resulting in a follow-up period of 3–9 years after NICU discharge. The local medical ethics review committee (Amsterdam UMC) deemed this study exempt (2024.1045), and consent for reuse of collected data was waived for the purpose of health care evaluation. This study was conducted in accordance with the Declaration of Helsinki.

The following characteristics were extracted from the electronic health records: GA (classified as extremely preterm (GA < 28 weeks) or very preterm (GA ≥ 28 weeks)), birthweight (BW), small for gestational age (BW < 10th percentile of Dutch reference curves), sex, moderate or severe BPD defined using the Bancalari definition [7], NEC grade 2 or higher, culture-proven sepsis, and intraventricular hemorrhage (IVH) grade 3 or higher [8]. All PICU admissions, length of admissions, and reasons for admission were collected. Reasons for admission to the PICU were categorized into respiratory, cardiovascular, sepsis/septic shock, neurological, gastrointestinal, renal, endocrine/metabolic, post-operative care, trauma, or other. Respiratory classification was further subcategorized into infection, wheeze/status asthmaticus, or other. Per local protocol, in our center, the decision to admit preterm children to the PICU postoperatively is based on current weight < 3 kg and/or need for supplemental oxygen, or is at the discretion of the attending physician depending on patient status and type of surgery.

### Statistical analysis

For descriptive statistics, dichotomous or categorical variables are reported as frequencies and percentages and continuous variables as medians with interquartile ranges (IQR). For graphical representation, Kaplan–Meier (KM) curves were constructed and depict the time to event, defined as (first) PICU admission. As we were not primarily focused on time-varying covariates and as there was very little variation in time-to-event captured by sufficient minimal follow-up time (3 years) after NICU discharge, we chose to perform a multivariable logistic regression model to assess which risk factors at patient level are associated with PICU admission and to adjust for possible confounding. Predefined predictor variables were sex, GA, SGA, NEC, IVH, and BPD, as based on previous literature and clinical relevance [2, 4, 5]. The outcomes are reported as odds ratios (OR) and 95% confidence intervals (CI). A *p*-value < 0.05 is considered statistically significant. More details on the statistical analysis can be found in the Supplemental material.

## Results

Among the 537 preterm infants who were admitted to the NICU during the study period, 78 (14.5%) died during their NICU stay, resulting in a study cohort of 459 patients. No patients born at GA < 30 weeks in our NICU were missed within this period. Demographics and baseline characteristics are summarized in eTable 1.

Among all included children, 50 patients (10.9%) had a total of 80 admissions to the PICU during follow-up, with admission rates varying by GA (eTable 2). The median (IQR) age at PICU admission was 4.0 (3.0–15.5) months, and the median (IQR) length of PICU stay was 2.0 (0.3–4.0) days (eTable 3). Thirty-three admissions (41.3%) were due to a primary respiratory illness and 41 admissions (51.3%) were due to post-operative care, mostly for elective surgery (eTable 3).

**Fig. 1:**
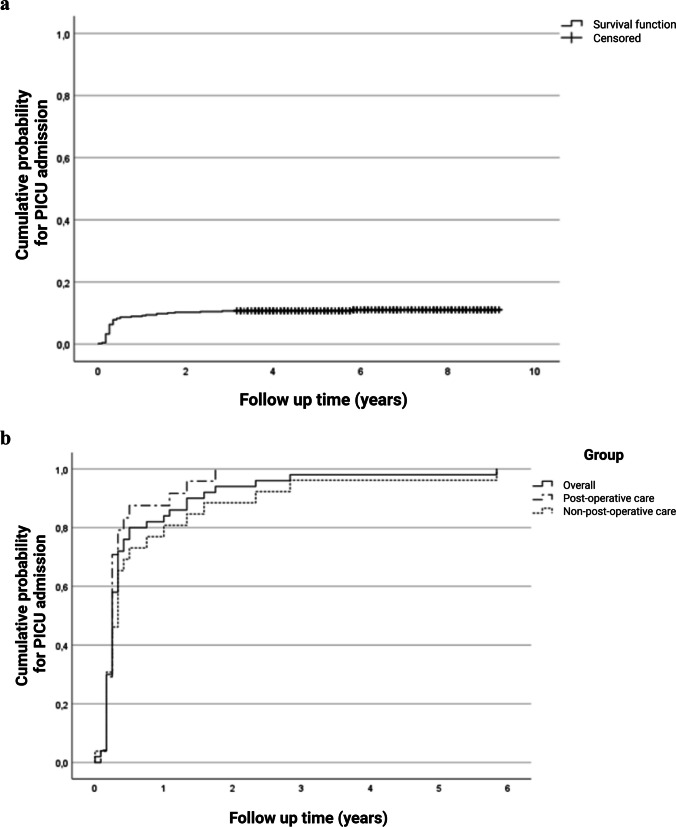
Inverse Kaplan–Meier (KM) curves showing the time (years) to event, defined as the first PICU admission after NICU discharge. The cumulative probability (**a**) for PICU admission for all infants included in this study, and (**b**) for the subgroup of infants with one or more PICU admission. The solid curve represents the overall KM curve. The other curves show the overall curve stratified by reason for admission: post-operative (dash-dotted) or non-post-operative (dotted). KM, Kaplan–Meier; PICU, pediatric intensive care unit; NICU, neonatal intensive care unit

Figure 1 depicts the cumulative probability of experiencing a PICU admission over time for all patients in this cohort (Fig. 1a), and for all patients with one or more PICU admissions, as well as for subgroups stratified by post-operative care and non-post-operative care admission reasons (Fig. 1b). The majority (94%) of the patients were admitted to the PICU for the first time before the age of 2 years.

For assessment of potential predictors, univariable analyses were performed (Supplementary material and eTable 4), showing that GA < 28 weeks and having diagnosis of NEC grade ≥ 2 or BPD were associated with PICU admission. To adjust for confounders, five predictors were included in the multivariable model to assess independent associations with PICU admission. This multivariable analysis confirmed that variables GA < 28 weeks and NEC grade ≥ 2, but not BPD, were independently associated with PICU admission in our cohort (Table 1). The Hosmer and Lemeshow test showed a *p*-value of 0.714, and the area under the curve (AUC) of the receiver operating characteristic (ROC) curve was 0.738.

**Table 1:** Multivariable prediction model for a PICU admission

Predictor		aOR	(95%CI)	*P*-value
Sex	Male	Reference	-	-
	Female	1.10	(0.58–2.08)	0.770
GA (< 28 weeks)		2.40	(1.13–5.11)	0.023
SGA (< 10^th^ centile)		1.63	(0.81–3.28)	0.172
NEC, Grade ≥ 2		5.82	(2.86–11.85)	< 0.001
BPD (moderate/severe)		1.40	(0.68–2.86)	0.363

*aOR* adjusted odds ratio, *CI* confidence interval, *GA* gestational age, *SGA* small for gestational age, *NEC* necrotizing enterocolitis, *BPD* bronchopulmonary dysplasia

## Discussion

In the current retrospective study, approximately 10% of preterm infants with a GA < 30 weeks were admitted at least once to the PICU within a few years after NICU discharge. Both GA < 28 weeks and NEC ≥ grade 2 were independently associated with PICU admission.

Survivors of preterm birth are at increased risk for long-term morbidity, which often leads to high health care utilization and costs [3, 9]. The current study adds to the literature by analyzing PICU admissions after NICU discharge, using a relatively long follow-up period of up to 9 years. So far, insight into this specific topic is relatively scarce, but important, since it may aid in organizing acute and follow-up health care services and allocating resources efficiently [10]. Furthermore, this research could help to identify parents/caregivers who are at increased risk for anxiety and post-traumatic stress symptoms (PTSS), particularly those affected by the conflicting experiences encountered in both NICU and PICU [11].

Our single-center results show an overall PICU admission rate of 10.9%, with rates varying according to GA. Considering the complex multimorbidity of preterm infants, this proportion may not appear to be very high at first glance. However, compared to an estimated general PICU admission incidence of approximately 100 per 100,000 (0.1%) in children of the same age range in the Netherlands [12], the risk for PICU admission among preterm infants is higher by two orders of magnitude. In addition, our study found a PICU admission rate almost twice as the one reported from a large cohort in the UK by van Hasselt et al. [2]. The difference in these findings might be due to the smaller sample size, the lower GA range, and the longer follow-up of our study. In addition, differences in health care organization such as admission thresholds, referral practices, and/or peri-operative protocols might have further contributed. Risk factors for PICU admission in our cohort were both GA and NEC, comparable to findings by van Hasselt et al. [2]. Independent of preterm birth, NEC may have gastro-intestinal, lung, and neurodevelopmental sequelae [13], rendering these children more vulnerable which may form an important driver for enhanced risk for PICU admission in later life. In the study by van Hasselt, BPD and brain injury were also independently associated with PICU admission. This was not confirmed in our study, potentially due to limited statistical power. A large part of PICU admissions in our study were due to respiratory illnesses. This finding is in line with emerging evidence for early, as well as lifelong, adverse lung function outcomes after preterm birth, not primarily connected to a diagnosis of BPD [14].

Importantly, half of the PICU admissions in our cohort were due to post-operative care, a proportion that is higher than reported by van Hasselt et al. (17.6%) [2]. By far, the majority of postoperative PICU admissions in our study were for elective surgery. Future research more specifically targeting such elective admissions may delineate whether PICU utilization in this subgroup is always justified, as there might be risk of overuse of PICU resources depending on local protocols.

Strengths of this study are the relatively long follow-up and the elaborate manual checking of patient records instead of relying on registry data. Limitations can be summarized as follows: (a) the retrospective design, which may have caused missed data on PICU admission and limited us to fully grasp whether decisions for postoperative PICU admissions were based on the local protocol or on patient/surgery status; (b) the relatively low number of events for the primary outcome, limiting the number of predictors in the multivariable analysis; (c) the single-center design, which limits generalizability of our findings to other centers or countries with different local protocols, admission thresholds, and referral and surgery practices. No other NICU or PICU serves the same geographical referral area, which encompasses 50 municipalities. However, if some patients moved out of this rather large region over time, any PICU admissions in another center will have been lost to follow-up. As we estimate, this may have slightly underestimated our findings by less than 5% (based on Statistics Netherlands data); we do not expect this will have led to meaningful impact.

In conclusion, in our study cohort, approximately one in ten preterm-born children (GA < 30 weeks) required at least one PICU admission after NICU discharge, with extreme prematurity and NEC being independent predictors. In particular, infants born < 28 weeks or with diagnosis of NEC ≥ grade 2 might benefit from enhanced surveillance or structured long-term follow-up pathways involving multidisciplinary teams. Further research into this specific, vulnerable patient group would be valuable for improving prognostication, prevention, and health care planning of long-term morbidity trajectories of these children and their families.

## Supplementary Information

Below is the link to the electronic supplementary material.

ESM 1 Supplementary Material 1 (DOCX 33.6 KB)

ESM 2 Supplementary Material 2 (DOCX 34.7 KB)

## Data Availability

No datasets were generated or analysed during the current study.

## Abbreviations

AUC: Area under the curve
AUMC: Amsterdam University Medical Center
BPD: Bronchopulmonary dysplasia
BW: Birthweight
CI: Confidence interval
GA: Gestational age
IQR: Interquartile range
IVH: Intraventricular hemorrhage
KM: Kaplan-Meier
NEC: Necrotizing enterocolitis
NICU: Neonatal intensive care unit
OR: Odds ratio
PICU: Pediatric intensive care unit
ROC: Receiver operating characteristic
SGA: Small for gestational age
VIF: Variance inflation factor
