# Supplementary material for: Risk for pediatric intensive care utilization in children born before 30 weeks of gestation: a single-center study
Source: Eur J Pediatr. 2026 Jan 19;185(2):90. doi: 10.1007/s00431-025-06714-4 (PMC12816077; doi:10.1007/s00431-025-06714-4)
Supplement: Supplementary file 2 — Supplementary Material 2 (DOCX 34.7 KB) [file 431_2025_6714_MOESM2_ESM.docx]

**Supplementary Material of article:**

**Risk for pediatric intensive care utilization in children born before 30 weeks of gestation: a Single-Center Study**

Annemijn C.C.B. Ekelmans^1,2,3,4^, Rosemarie de Ridder^1,4^, Suzanne W.J. Terheggen^2,4^, Marieke H. Otten^3,4^, Anton H.L.C. van Kaam^1,4^, Job B.M. van Woensel^3,4^, G.J. Hutten^1,4^, Reinout A. Bem^3,4^

^1^ Neonatal Intensive Care Unit, Emma Children’s Hospital, Amsterdam University Medical Centers, location University of Amsterdam, Amsterdam, The Netherlands

^2^ Department of Pulmonology, Emma Children’s Hospital, Amsterdam University Medical Centers, location University of Amsterdam, Amsterdam, The Netherlands

**^3^** Pediatric Intensive Care Unit, Emma Children’s Hospital, Amsterdam University Medical Centers, location University of Amsterdam, Amsterdam, The Netherlands

^4^ Amsterdam Reproduction & Development research institute, Amsterdam, the Netherlands

**Corresponding author:** Annemijn C.C.B. Ekelmans ([a.c.c.b.ekelmans@amsterdamumc.nl](mailto:a.c.c.b.ekelmans@amsterdamumc.nl))

**SUPPLEMENTARY INFORMATION**

**Supplementary eTable 1** Demographics and baseline characteristics

| Demographic variables |  |
| --- | --- |
| Total, n(%) | 459 (100) |
| Female, n(%) | 208 (45.3) |
| Gestational Age, (weeks)^a^  GA < 28 weeks, n(%)  GA ≥ 28 weeks, n(%) | 27.9 (26.3-28.9)  247 (53.8)  212 (46.2) |
| Birthweight, (g)^a^ | 975 (820-1205) |
| Small for gestational age^b^, n(%) | 129 (28.1) |
| Singleton, n(%) | 325 (70.8) |
| Caesarean section, n(%) | 204 (44.4) |
| Duration of NICU admission (days)^a^ | 29 (15.0-51.0) |
| BPD, n(%)  Moderate BPD  Severe BPD | 9 (2.0)  98 (21.4) |
| NEC ≥ grade 2, n(%) | 57 (12.4) |
| IVH ≥ grade 3, n(%) | 14 (3.1) |
| Sepsis, n(%) | 151 (32.9) |

^a^Reported as median (IQR), ^b^SGA defined as birthweight < 10^th^ percentile of Dutch reference curve

GA: gestational age. SGA: small for gestational age. NICU: neonatal intensive care unit. BPD: bronchopulmonary dysplasia. NEC: necrotizing enterocolits. IVH: intraventricular hemorrhage.

**Supplementary eTable 2** Number and proportion of patients with ≥ 1 PICU admission by gestational age

| GA, weeks | ≥ 1 PICU admission, n (% of admissions in category) |
| --- | --- |
| 24 (n = 34) | 8 (23.5) |
| 25 (n = 57) | 12 (21.1) |
| 26 (n = 80) | 12 (15.0) |
| 27 (n = 76) | 5 (6.6) |
| 28 (n = 105) | 6 (5.7) |
| 29 (n = 107) | 7 (6.5) |
| Total (n = 459) | 50 (10.9) |

GA: gestational age. PICU: pediatric intensive care unit.

**Supplementary eTable 3** Characteristics of PICU admissions among all patients with ≥ 1 admission

| Characteristics |  |
| --- | --- |
| Total patients with PICU admission, n(% of cohort) | 50 (10.9) |
| Total PICU admissions, n | 80 |
| PICU admissions per patient^a^ | 1 (1.0-2.0) |
| Age at PICU admission (months)^a,c^ | 4 (3.0-15.5)^d^ |
| Duration of PICU admission (days)^a^  Post-operative care (days)^a^  Non-post-operative care (days)^a^ | 2 (0.3-4)  1 (0.0-2.5)  3 (2.0-8.0) |
| PICU mortality, n | 0 |
| Reason for PICU admission, n(% of total admissions)  Respiratory  Infection  Wheeze/status asthmaticus  Other  Sepsis/septic shock  Neurological  Gastrointestinal  Endocrine/metabolic  Post-operative care  Elective  Non-elective  Other^b^ | 33 (41.3%)  22 (27.5)  3 (3.8)  8 (10.0)  1 (1.3)  2 (2.5)  2 (2.5)  1 (1.3)  41 (51.3)  36 (45.0)  5 (6.3)  0 (0) |

^a^Reported as median (IQR)

^b^Including the reasons; cardiovascular, renal, trauma, and other.

^c^Age at PICU admission for all PICU admissions (n=80); ^d^ full range: 0-74 months.

**Supplementary material for Methods – statistical analysis**

Both univariable (eTable 4) and multivariable (Table 1) logistic regression were performed. The maximum number of predictors for the multivariable model was established as 10% of the number of events, as defined by the first PICU admission. According to this 10%-rule, only five predictors were allowed in the multivariable model as we observed n=50 events (50 patients with at least one PICU admission). Since IVH had a low incidence, only sex, GA, SGA, BPD and NEC were included in the final model. Multicollinearity was checked by conducting a correlation matrix and determining variance inflation factor (VIF). Finally, to assess model fit, a receiver operating characteristic (ROC) curve and Hosmer & Lemeshow test were performed. By convention we deemed an area under the curve (AUC) for the ROC of > 0.7 acceptable. For statistical analysis and computing, SPSS version 28.0 (IBM Corp., Armonk, NY, USA) was used.

**Supplementary eTable 4** Univariable prediction model for PICU admission. The variables GA, NEC and BPD demonstrate a P-value < 0.05

| predictor |  | OR | 95%CI | P-value |
| --- | --- | --- | --- | --- |
| Sex | Male  Female | Reference  0.942 | -  0.52 – 1.70 | -  0.843 |
| GA (< 28 weeks) |  | 2.70 | 1.39 – 5.22 | 0.003 |
| Sga (< 10^th^ centile) |  | 1.23 | 0.66 – 2.32 | 0.517 |
| NEC, grade ≥ 2 |  | 6.70 | 3.47 – 12.96 | < 0.001 |
| bpd (moderate/severe) |  | 3.00 | 1.63 – 5.50 | < 0.001 |

OR: odds ratio. CI: confidence interval. GA: gestational age. SGA: small for gestational age. NEC: necrotizing enterocolits. BPD: bronchopulmonary dysplasia.
